# Supplementary material for: Causal Relationship between Aging and Anorexia Nervosa: A White-Matter-Microstructure-Mediated Mendelian Randomization Analysis
Source: Biomedicines. 2024 Aug 16;12(8):1874. doi: 10.3390/biomedicines12081874 (PMC11351342; doi:10.3390/biomedicines12081874)
Supplement: Supplementary file 1 [file biomedicines-12-01874-s001.zip › Supplementary Figure/Figure S4/Singe SNP plot of MR analysis for TL to ubm-b-1529.pdf]

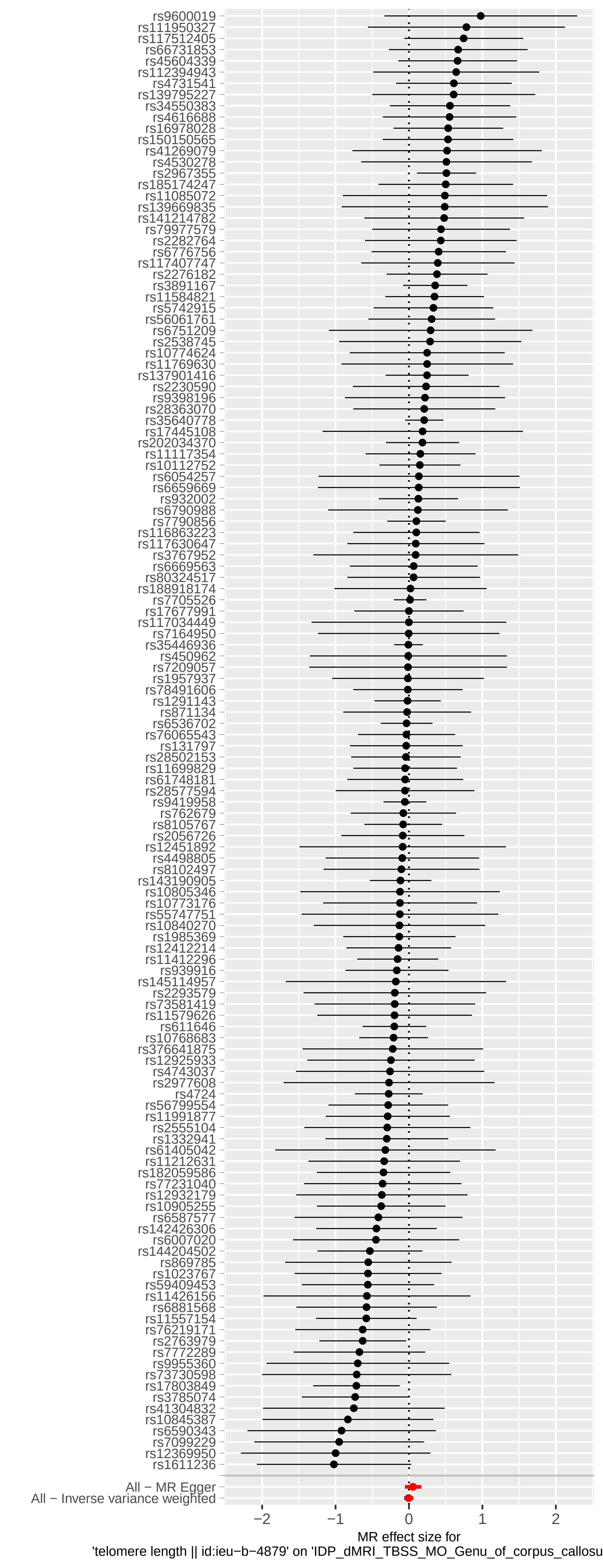
rs9600019
rs111950327
rs117512405
rs66731853
rs45604339
rs112394943
rs4731541
rs139795227
rs34550383
rs4616688
rs16978028
rs150150565
rs41269079
rs4530278
rs2967355
rs185174247
rs11085072
rs139669835
rs141214782
rs79977579
rs2282764
rs6776756
rs117407747
rs2276182
rs3891167
rs11584821
rs5742915
rs56061761
rs6751209
rs2538745
rs10774624
rs11769630
rs137901416
rs2230590
rs9398196
rs28363070
rs35640778
rs17445108
rs202034370
rs11117354
rs10112752
rs6054257
rs6659669
rs932002
rs6790988
rs7790856
rs116863223
rs117630647
rs3767952
rs6669563
rs80324517
rs188918174
rs7705526
rs17677991
rs117034449
rs7164950
rs35446936
rs450962
rs7209057
rs1957937
rs78491606
rs1291143
rs871134
rs6536702
rs76065543
rs131797
rs28502153
rs11699829
rs61748181
rs28577594
rs9419958
rs762679
rs8105767
rs2056726
rs12451892
rs4498805
rs8102497
rs143190905
rs10805346
rs10773176
rs55747751
rs10840270
rs1985369
rs12412214
rs11412296
rs939916
rs145114957
rs2293579
rs73581419
rs11579626
rs611646
rs10768683
rs376641875
rs12925933
rs4743037
rs2977608
rs4724
rs56799554
rs11991877
rs2555104
rs1332941
rs61405042
rs11212631
rs182059586
rs77231040
rs12932179
rs10905255
rs6587577
rs142426306
rs6007020
rs144204502
rs869785
rs1023767
rs59409453
rs11426156
rs6881568
rs11557154
rs76219171
rs2763979
rs7772289
rs9955360
rs73730598
rs17803849
rs3785074
rs41304832
rs10845387
rs6590343
rs7099229
rs12369950
rs1611236
All − MR Egger
All − Inverse variance weighted
−2
−1
0
1
2
MR effect size for
'telomere length || id:ieu−b−4879' on 'IDP_dMRI_TBSS_MO_Genu_of_corpus_callosu
